# Novel Covalent Modifier-Induced Local Conformational Changes within the Intrinsically Disordered Region of the Androgen Receptor

**DOI:** 10.3390/biology12111442

**Published:** 2023-11-17

**Authors:** Michael T. Harnish, Daniel Lopez, Corbin T. Morrison, Ramesh Narayanan, Elias J. Fernandez, Tongye Shen

**Affiliations:** 1 Department of Biochemistry & Cellular and Molecular Biology, University of Tennessee, Knoxville, TN 37996, USA; mharnish@vols.utk.edu (M.T.H.); dlopez7@vols.utk.edu (D.L.); cmorri80@vols.utk.edu (C.T.M.); elias.fernandez@utk.edu (E.J.F.); 2 Department of Medicine, College of Medicine, University of Tennessee Health Science Center, Memphis, TN 38103, USA; rnaraya4@uthsc.edu

**Keywords:** intrinsically disordered protein, covalent drug, protein conformation, androgen receptor, molecular dynamics simulation

## Abstract

**Simple Summary:**

The androgen receptor (AR) is an important protein involved in sensing hormones. The over-activation of a class of AR can lead to cellular dysfunction and diseases such as prostate cancer. This malfunction is connected to abnormal protein clusters known as condensates. To address this, a series of novel small molecules, which can potentially dissolve AR condensates, have been tested for their ability to change the shape of the AR. These molecules interact with disordered regions of the AR and consequently, weak structural signatures are buried in a vast amount of seemingly random shapes of the AR. We use biophysical models and computational approaches to examine the shapes of the AR when it is bound to a therapeutically promising small-molecule modulator. Here, we provide insights on how subtle alterations of the small-molecule modulator can strongly affect their ability to alter AR shapes.

**Abstract:**

Intrinsically disordered regions (IDRs) of transcription factors play an important biological role in liquid condensate formation and gene regulation. It is thus desirable to investigate the druggability of IDRs and how small-molecule binders can alter their conformational stability. For the androgen receptor (AR), certain covalent ligands induce important changes, such as the neutralization of the condensate. To understand the specificity of ligand–IDR interaction and potential implications for the mechanism of neutralizing liquid–liquid phase separation (LLPS), we modeled and performed computer simulations of ligand-bound peptide segments obtained from the human AR. We analyzed how different covalent ligands affect local secondary structure, protein contact map, and protein–ligand contacts for these protein systems. We find that effective neutralizers make specific interactions (such as those between cyanopyrazole and tryptophan) that alter the helical propensity of the peptide segments. These findings on the mechanism of action can be useful for designing molecules that influence IDR structure and condensate of the AR in the future.

## 1. Introduction

A current area of focus in understanding gene regulation is the function of intrinsically disordered proteins (IDPs) and modular proteins containing intrinsically disordered regions (IDRs) [1,2]. About 10% of eukaryotic proteins are suspected to be fully disordered, and 40% contain a disordered loop region of at least 50 amino acids [3]. These IDRs are identified in nearly all nucleotide-interacting proteins, including various transcriptional factors [4] and RNA-binding proteins [5,6].

How IDPs and IDRs function for regulatory systems is not well understood. While they are known to have essential biological roles in regulation, and mutations within such regions lead to malfunction, they are demonstrated to be predominantly structureless ensembles that do not have well-defined, unique conformational folds. For instance, the N-terminal domain (NTD) of the androgen receptor (AR) may play a major role in gene activation [7,8], including the initiation of one aggressive form of prostate cancer: castration-resistant prostate cancer (CRPC) [9,10,11]. While the mechanism of IDR function might not be universal, the IDRs of the AR can induce liquid–liquid phase separation (LLPS) and protein-rich transcriptomic condensate [12]. Aggregates of AR exhibit enhanced interactions between the AR and coactivators which might be a reason for the excessive stimulation of this transcription factor [13]. It is thus interesting to study how one can suppress such aggregation and LLPS using small molecules [14].

Drug specificity typically arises through the recognition of specific structural features of the target protein, making the specificity of small molecules on IDR activity intriguing. One example of actions by small molecules on IDR systems is the neutralization of a class of condensates by 1,6-hexanediol [15,16], while its isomer, 2,5-hexanediol, is less effective [17]. As researchers begin to establish experimental and computational methods that characterize IDP conformational ensembles, it would be beneficial to understand how these structural ensembles can be affected by small molecules. Commonly used experimental methods, such as circular dichroism and hydrodynamic radius studies, can provide data about certain bulk features of the system. However, they do not provide structure details at per residue resolution. Additionally, single molecule studies such as FRET can be costly to apply when there is a need for monitoring many structural details and comparing different perturbations [18,19,20,21]. The lack of well-characterized structures also makes studying protein–ligand interactions more difficult for computational methods. It is challenging to apply molecular docking and other conventional methods, which require well-defined native protein conformation(s) [22,23]. Instead, we choose to use all-atom molecular dynamics simulations to directly explore the conformations with and without the ligand present in an atomistic description. 

In this work, we focus on examining how the human AR-NTD interacts with a specific set of designed covalent ligands. Specifically, we are motivated by the fact that a small-molecule modifier can dissolve the AR condensate with specificity [24,25]. The AR is an important member of the nuclear receptor (NR) family, all of which are ligand-activated, multi-domain transcription factors [26,27,28]. The N-terminal domain (NTD) is highly varied among the members of the NR family, which is often not structured and the least studied domain [8]. The DNA-binding domain (DBD) is the domain that directly binds DNA to initiate gene transcription. Finally, the ligand-binding domain (LBD) contains a binding pocket, which activates the NR upon ligand binding. The ways in which these nuclear receptors sense the ligand, different domains communicate, and the ways in which they activate gene transcription have posed many important questions that have practical applications in drug discovery [29]. 

Typically, the efforts of designing antagonists and inverse agonists of NRs are focused on the LBD. Both ligand-dependent and ligand-independent activation are extremely important for the AR, and suppression of the receptor activity has become one common prostate cancer therapy [30]. A class of AR antagonists, such as enzalutamide, have been established to interact with LBD [31]. A splice variant of AR associated with CRPC lacks the LBD, and it renders drugs such as enzalutamide ineffective; however, it is striking that this mutant is more active than wild-type agonist-bound AR. The largely disordered NTD region [13] is suggested to be responsible for this overstimulation, since this extreme form of constitutive activity comes from androgen-independent activation via the presumed AR-NTD activation function 1 (AF1)–coactivator interaction [9,32]. In recent years, there have been sustained and significant efforts to design IDR-interacting ligands that inactivate the AR [10,16,25,33]. 

One difficulty of studying ligand–IDR interaction is the absence of information on specific interactions between protein and ligand. A breakthrough was reported recently that a set of ligands are able to interact with this IDR and result in the dissociation of AR aggregates and liquid–liquid phase separation (LLPS) [12]. These ligands are termed Selective AR irreversible covalent antagonists (SARICAs) and share a common theme of a dual-ring (A–B) structure. The covalent nature of SARICAs is significant from the structural biology viewpoint, since these covalent modifiers [34,35] contain a reactive functional group and interact with proteins more strongly than their traditional, noncovalent counterparts. Earlier studies of noncovalent ligands (such as UT-34) have shown their antagonistic potentials, but ways in which these ligands can specifically interact with the IDR has been difficult to assess [24]. Since the newly reported covalent ligands are cysteine linked, the location of protein–ligand interaction can be determined explicitly. 

In this study, we constructed the peptide systems with and without covalent ligands at an atomistic resolution. From a set of simulations, we can sample ways in which the peptide conformations are affected by ligands and provide insight on structural changes upon ligand interaction. The results can lead to a better understanding of IDR–ligand interaction and improved, rationally designed SARICAs in the future. Our simulation results indicate that effective drug molecules have strong effects on altering local secondary structures. Specifically, the cyanopyrazole (B-ring) of SARICAs seems to be responsible for reducing helical propensity. Also, these effective drugs dissolve the condensate, which suggests that the condensate might be stabilized by interactions involving these structured helical components. This work demonstrates how drug molecules can affect the behavior of IDRs within the AR-AF1 with specificity. Although our observations may be a product of the specific sequence of human AR, the conclusion might be applicable to systems with similar characteristics, such as the glucocorticoid receptor [36] and some other transcriptional factors. Although IDRs of different proteins may be quite different in their nature, this work can provide a concrete example of how small chemicals can influence the conformations of this type of IDRs. 

## 2. Systems and Method

### 2.1. Selection of Peptide Segments and Ligands

Ideally, one would directly sample the entire set of IDR conformations and study how various covalent ligands affect AF1 conformations and associated aggregates. However, these large, disordered protein systems (300+ a.a. residues) would be challenging to adequately sample in straightforward atomistic molecular dynamics simulation in an explicit-solvent setup [37] and thus, advanced sampling methods and/or special features of the system should be exploited for IDR–ligand interaction and IDR sampling [38,39]. The intact NTD of the human AR is quite large when we compare it to other members of the NR family [40]. It has about 560 residues and contains the AF1 region (141–486) which is considered a transactivation region. The sequence information of AR comes from UniProt ID P10275. The whole AF1 region is used for studying drug effects in the LLPS experiment [12]. Motivated by experimental findings, we focus on constructing and sampling important NTD peptide segments and we address how drug molecules (SARICAs) affect local structures of the NTD segments.

**Figure 1:**
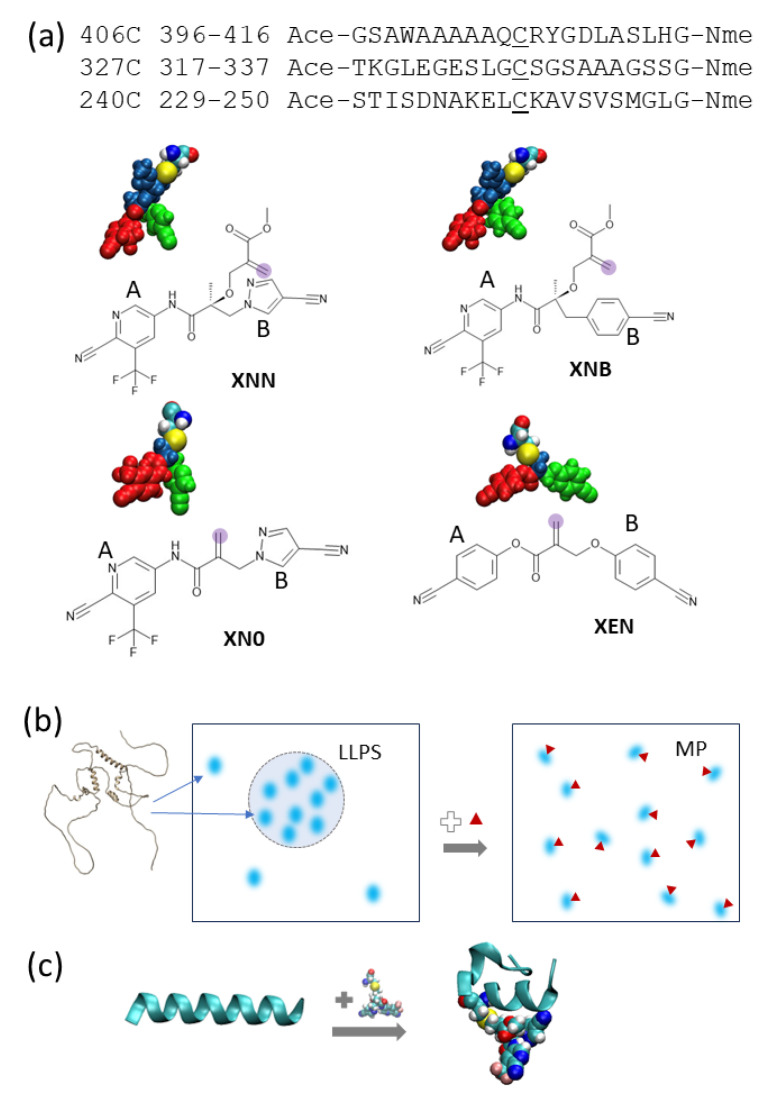
(**a**) Three peptides from the AF-1 region of AR are used for this study. Each peptide contains 21 amino acid residues centered at a Cys residue and blocked at the ends by ACE and NME. The four ligand-attached amino acid residues (XNN, XNB, XN0, and XEN) are shown in the 3D representation. Each one is a Cys with its thiol group covalently bonded with a specific ligand. Three different colors on the residue indicate A-ring (red), B-ring (green), and stem (navy) fragments. The reactive carbon of each covalent ligand is shaded in purple. The A and B rings of the ligands are labeled. (**b**) A cartoon illustration of how liquid–liquid phase separation (LLPS) can be affected by small-molecule binder (red triangles) where AF-1 (fuzzy blue dots) changes interaction patterns and becomes a monophase protein solution. A hypothesis on the underlying mechanism (structure changes induced by ligand) is being tested. (**c**) Illustration of the effect of SARICA on peptide conformation from simulation snapshots (conformations selected from unliganded and XNN-bound peptide 406C, left and right, respectively).

The three peptide sequences chosen for this study are shown in Figure 1. Their position in the full sequence of AF1 is shown in Supplementary Material Figure S1. Although it is well known that the NTD of nuclear receptors is considered a disordered protein domain in general, bioinformatics analysis and biophysical studies present a complex picture for the AR NTD. The Predictor of Natural Disordered Regions (PONDR) [41] shows a highly varied degree of order/disorder across AF1 [42], as shown in Supplementary Material Figure S1. Three types of low complexity IDR signatures [43] (polyproline, polyglycine, and polyglutamate) are all present in AF1. It is likely that the structure of AR-AF1 is a mixture of transient helical segments and disordered regions [44]. The 3D structure predictor AlphaFold2 [45] also presents a picture of human AF1 alone as a largely disordered domain with 3 to 4 partially structured (helical) regions loosely packed at its core, as shown in Supplementary Material Figure S2. A similar picture is presented for mouse AF1 with a conserved region showing nearly identical conformations. NMR studies have also indicated that the tau5 region of AR-AF1 (330–450) has three partially ordered segments [33,46]. Of the 8 Cys residues in the human AR-AF1 region, residues C406 and C327 have been identified as the locations where the SARICAs preferentially ligate, and residue C406 is noticeably modified in mass spectrometry studies [12]. We used notation “C406” for the residue and “406C” for the corresponding peptide segment throughout this work. Based on these findings, two segments centered on these two residues were used for this study. A third segment centered on C240, which is highly conserved among AR of various species, was chosen for a comparison. We constructed 21-residue long peptide segments centered at each of these Cys residues. These peptides allowed us exploration of how the ligand-attached residue affects the residues within a span of 10 residues. Longer segments may include more nonlocal interactions; however, they increase the sampling difficulty exponentially. The commonly used capping residues ACE [−C(=O)−CH_3_] and NME [C(= O) − NH −CH_3_] were added to block the N- and C-termini, respectively, which prevent the terminal zwitterion effects.

As shown in Figure 1, we constructed four modified cysteine residues that are covalently bound to distinct ligands: XNN, XNB, XN0, and XEN. We began with XNN, which is a cysteine residue attached to UT-143, a modifier studied by Thiyagarajan et al. [12]. UT-143 has a pyridine–cyanopyrazole (A–B ring) structure with a linker segment extending from its backbone hydroxyl group to allow covalent binding; it was shown to be effective in vitro for the destruction of LLPS of the AR-NTD. While UT-143 was found to be the most effective, that study also examined three other ligands, two of which were ineffective (UT-153, NB-Enob) and one which was moderately effective (UT-215). Each of these molecules have multiple structural differences compared to UT-143, notably changes to A/B rings and the length of the linker. To isolate observed effects to specific structural components, we built XNB and XN0 as modified versions of XNN. XNB has a modified B ring which is changed from cyanopyrazole to a cyanophenyl group to match that of NB-Enob. Finally, XN0 is identical to XNN in terms of A/B rings, but it does not have a linker segment between the ligand backbone and the covalent binding site. We also modeled XEN, which contains cyanophenyl groups for both A- and B-rings, to verify the extent of A-ring involvement.

### 2.2. Simulation Setup and MD Procedure

AMBER force field 99SB [47] was selected for peptide modeling. TLEAP from AMBER tools [48] was used to construct peptides using sequence-based builder. After adding terminal blocking residues, solvating with TIP3P waters, and adding counter ions (if necessary), each unmodified peptide system contained roughly 3000–6000 atoms. We examined two initial conformations: α-helix and full extended β-strand, where we labeled the systems “H” and “E”, respectively. 

For covalent ligands, the 3D structure was constructed manually. The chiral carbon of the ligand was set to a specific value reported in stereo SMILE code (Supplementary Material Figure S3). We note that in the experiments, the specific chirality of one linkage carbon cannot be controlled easily and is unlikely to be stereochemically pure. We assumed that the chirality choice is inconsequential for this study and thus picked one chirality. For obtaining covalent ligand force field parameters, we went through a procedure that is similar to that of a post-translational modification of amino acid residues. We first constructed the 3D structure model of the modified, ligand-bound cysteine residues. We then went through quantum chemistry calculations using the ANTECHAMBER module with the AM1-BCC charge model. Next, we went through the PREPGEN module and checked missing force field parameters using PRMCHK2. We carefully inspected line items in .ac files, especially those referencing backbone atoms that might need a direct correction of their chemistry. This whole procedure resulted in ligand-attached cysteine residues that are ready to be used as alternative building blocks for constructing polypeptides.

We initially performed 55 ns MD simulations for each peptide system. We took the first 5 ns as an equilibration run and did not use it for the 50 ns analysis. We performed simulations for an additional 50 ns for some of the systems to check the convergence. The full list of systems is shown in Table 1, where a total of 14 systems were constructed to compare various aspects of the protein–ligand interaction and peptide structural stability. Snapshots were taken at 1 ps intervals during simulation, although certain analyses (contact map and protein–ligand contacts) were performed at a lower frequency of per 10 ps. Two types of information were examined: secondary structure and contact interaction. For secondary structure information, we used stacked bar graphs to show how the ligands affect the local secondary structures of the peptides. We also used residue–residue contact heatmaps to display contact interactions. For ligands, we subdivided them into 3 parts so we could study the specificity of the individual components.

### 2.3. Analysis of Conformations

Once simulation trajectories were obtained, several types of analyses were performed including local secondary structure, residue–residue contact heatmap, and statistical analysis of the conformational ensemble. The residue-resolved local secondary structure was computed at the interval of 10 ps. For each snapshot, computer program PTRAJ was used to provide the DSSP classification [49] of secondary structures and labeled each residue one of eight possible states: three α-helical structures, two β-strand structures, turn, bend, and none. The aggregated information on all frames in a trajectory can be displayed in a stacked bar graph. 

Contact maps are another way of characterizing protein and peptide conformations. A residue–residue contact is considered formed (contact value 1 assigned) between residues j and k when any atoms of residue j is within a distance cutoff (4.2 Å in this case) of any atoms of residue k [50]. Otherwise, the contact value is 0 and the contact is not formed. Concrete examples of how the result is dependent on the cutoff parameter can be found in [51] and in SI of ref [52]. After enumerating all j–k pairing and presenting the data in a contact matrix format, a contact map for each snapshot can be obtained. The mean contact map (heat map) is an ensemble average of the individual contact maps. As argued previously, contact degrees of freedom provide a useful alternative for describing conformations—especially for IDP conformations, conformational switches, and folding [39,53,54].

A more quantitative cross-comparison between the conformations of ligand-bound peptide conformations is using abstract (and collective) degrees of freedom (DOFs) to project the conformations to a low dimensional subspace. Since the number of contacts is large, statistical analysis should be used to compress data [55] further in order to facilitate visualization and cross-comparison between conformations of different ensembles. In this study, we applied the commonly used analysis method Uniform Manifold Approximation and Projection for Dimension Reduction (UMAP) [56] to the contact DOFs. UMAP can be conceptualized as a nonlinear representation of conformations with its transformed collective DOFs grounded in global conformation similarity. To provide a comparison, we also applied Principal Component Analysis (PCA) [57], which is traditionally used in finding collective modes of protein dynamics [58,59], to contact DOFs [50,53]. Eventually, either method can transform the contacts to new coordinates and describe conformations (the so-called conformation projection) using newly constructed collective DOFs.

## 3. Results and Discussion

**Figure 2:**
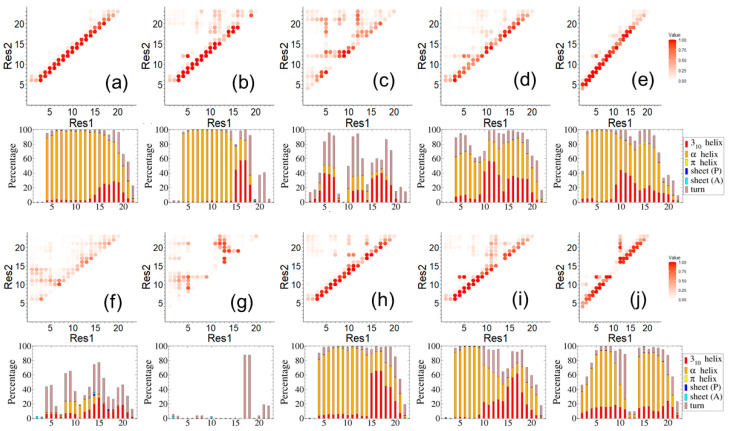
A comparison of peptide fold shown in contact maps and local secondary structures shown in stacked bar graphs for systems: (**a**) NTD_4H_1, (**b**) XNN_4H_1, (**c**) XN0_4H_1, (**d**) XNB_4H_1, (**e**) XEN_4H_1, (**f**) NTD_4E_1, (**g**) XNN_4E_1, (**h**) NTD_4H_2, (**i**) XNN_4H_2, and (**j**) XNN_3H_1. Here, “_1” and “_2” indicate the first and second 50 ns simulation, respectively.

We first examined the conformations at the local and secondary structure level using a contact map (Figure 2). This enabled multiple cross-comparisons that can be grouped into four aspects: ligand effects (unmodified vs. ligand attached), starting conformation influence (helical vs. extended), simulation length (first and second 50 ns), and specific peptide-ligation site (C406, C327, or C240).

As shown in Figure 2a–e, five simulations of peptide 406C with different ligand states were compared: the native peptide (Figure 2a) and four different ligand-attached systems: XNN (Figure 2b), XN0 (Figure 2c), XNB (Figure 2d), XEN (Figure 2e). The starting peptide conformation in all five cases was α-helical and the simulation was 50 ns. We observed that NTD_4H_1 (Figure 2a) largely maintains helical secondary structure and does not venture into other conformations. However, two simulations, XNN_4H_1 (Figure 2b) and XN0_4H_1 (Figure 2c), explored multiple conformations during the 50 ns sampling. Furthermore, the peptide was no longer helical, especially the C-terminal half when bound to each of the two ligands, XNN and XN0. Conversely, XNB_4H_1 and XEN_4H_1 appeared to retain noticeable levels of helical conformation. Earlier experimental results indicated that XNN and XN0 are likely to be effective LLPS neutralizers compared to XNB [12]. Our data suggests that structural destabilization induced by the cyanopyrazole ring might be a mechanism of LLPS neutralization. 

We mostly provided the ensemble-averaged properties here, but it is interesting to track the detailed dynamic information on the helix breakage, which we present in Supplementary Material Figure S4. Here, we observe that the peptide responds to ligand perturbation within 20 ns. A typical simulation can easily cover the timescale of helix formation dynamics (~1 ns) of extremely short peptides [60]. Certainly, the helix–coil transition might happen on a longer timescale for a larger, well-structured and complex protein than the simulation timescale used here [61]. Nevertheless, the simulation approach is well-suited to study helix–coil transition, especially for small peptides [62,63]. It should be noted that typical molecular dynamics simulation methods are used as sampling tools and do not necessarily have a one-to-one correspondence to real time [64]. It is known that simulation time can be faster than real time when the energy landscape of the force field (including solvent) is smoother than reality [65]. Additionally, the transient nature of IDR might also contribute to the fast dynamics we observed in simulation.

Since the starting conformations affect the finite-time sampling of molecular dynamics at the 50 ns timescale, we also showed the 406C peptides (extended start) in Figure 2f,g which is a direct comparison to the 406C peptides (helical start) shown in Figure 2a,b. We observed that the simulation of native peptide with extended start (NTD_4E_1) slowly transitions into helical structures, while simulation XNN_4E_1 does not form helical conformations during the same time span and remains almost entirely unstructured. We note that that the starting conformation did not affect the trend of observable conformational changes. Both showed native 406C with helical start remains helical while the equivalent extended start system trended towards helical conformation. 

In panels (Figure 2h,i), we displayed the second 50 ns results, which is a direct comparison of panels (Figure 2a,b) where the first 50 ns results are shown for NTD_406H and XNN_406H. The similarity between (Figure 2i,b) for the level of convergency for XNN_406H system can be observed. On the other hand, the native 406C starts to explore more non-helical folds during the second 50 ns. 

In addition to the segment 406C, we also studied peptide segments 327C and 240C. The residue C327 was previously reported to be susceptible to covalent bonding, though to a lesser extent than C406, whereas C240 was not involved in the ligand attachment and thus it provided us with a reference system. During the simulations, native peptide segments 327C did not maintain the α-helical structure and were largely disordered while peptide segments 240C maintained α-helical structure, but at a lesser level compared to 406C. In Figure 2j, we show the results of segment 327C with SARICA attached, specifically how XNN affects this segment with helical structure as the starting point. It can be observed that the effect of XNN on 327C is similar to that with 406C (Figure 2b), especially on how the ligand breaks the starting helical structure and how it interacts with the second half of the peptide segment. The simulations of chemical-attached segments 327C and 240C (both with helical start) showed that they quickly transition into a disordered state while the native ones stay relatively longer. If the starting conformation is fully extended, the segment centered on 327C remains largely disordered throughout the simulation. 

**Figure 3:**
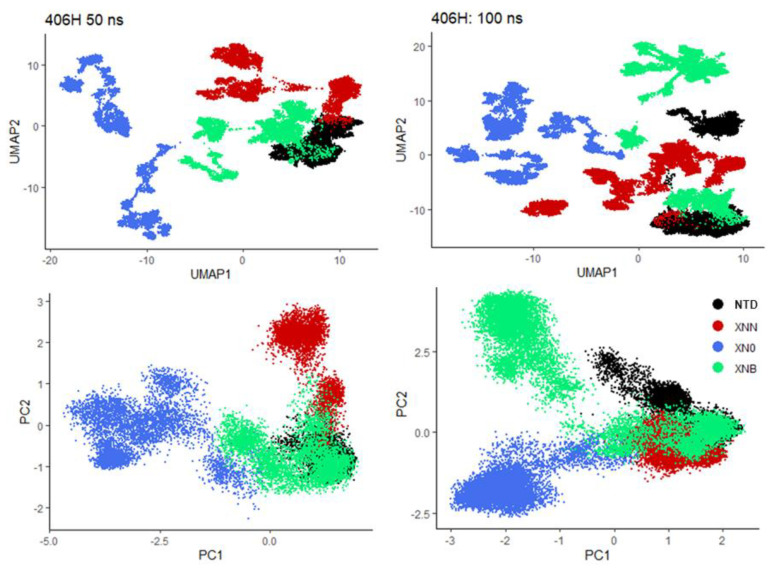
Peptide conformations expressed by contact interaction patterns are transformed into collective degrees of freedom (DOFs) using analysis tools, UMAP (**upper panels**) and PCA (**lower panels**). The first 50 ns results are shown on the left panels and the 100 ns results on the right.

For conformational dynamics, we display both UMAP and PCA projections in Figure 3. The raw DOFs are contacts, where each contact map of size 23 times 23 contributes a total of 253 nonredundant DOFs. A consistent pattern emerges as native 406C (first 50 ns) and the ineffective XNB both keep a similar conformation, whereas the effective XNN and XN0 systems explore distinct nonhelical conformations during the first 50 ns simulation (Figure 3, left panels). The corresponding right panels (Figure 3) include an additional 50 ns (total 100 ns) results. A comparison can be made with the left panels from which it can be observed how conformational dynamics evolve. We note that the right panel presents a slightly more complex picture of the protein dynamics which is understandable as both collective coordinates and projection values are different when varied amounts of sampling points are used. The UMAP coordinates are more sensitive than those of PCA, although both present a consistent picture that during the second 50 ns additional conformations lead to complex coordinates and complicated projections. We also observed that the second half of the 100 ns XNB simulation exhibited significant changes while the other systems were largely consistent. We did not think these ligand-attached peptide segments were fully sampled at this timescale; nevertheless, the results demonstrated how relatively easy it is for confirmations to escape the initial (helical) structures with the help of SARICAs. 

**Figure 4:**
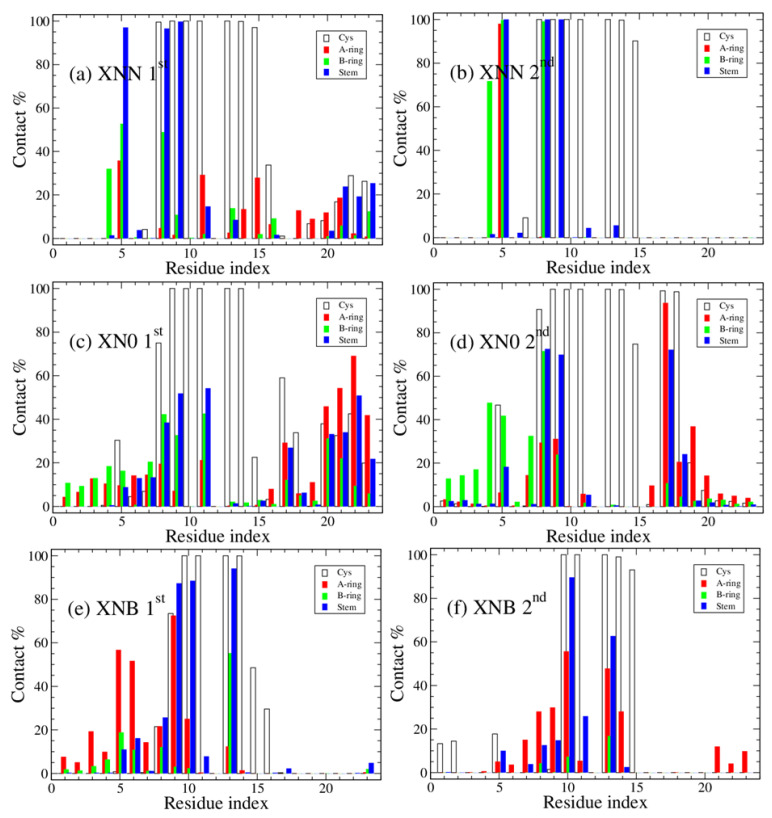
The percentage of ligand contact formation (contact ratio × 100%) during simulation is shown as a function of the residue index. The breakdown of the protein–ligand interaction patterns by fragments: Cys residue (empty), ligand A-ring (red), ligand B-ring (green), and ligand stem (navy). The three systems are peptides 406C centered with different ligands, XNN-4H, XN0-4H, and XNB-4H. The first and second 50 ns block results are shown on the left and right panels, respectively.

One important discovery of the previous experimental work on AR ligand specificity is that the cyanopyrazole (B-ring) is an effective LLPS neutralizer, whereas the cyanophenyl ring appears to be ineffective. To address B-ring specificity and specific protein–ligand interaction, we display the residue–ligand fragment contact interaction in Figure 4. Here, the contact between specific parts of a ligand and residue is shown. We can observe that the main residues that form contact between the B-ring of XNN and the peptide are around residues W399 and A403 (Residue #5 and Residue #9, using the internal index starting with capping residue and Cys is Residue #12), and it is likely that these peptide–ligand contacts disrupt the internal interaction of the native peptides. Similarly, the top contacts between the B-ring of XN0 are at nearly identical positions. In contrast, the top contacts between a different B-ring, that of XNB, and peptide are both low in comparison and at different locations. Therefore, we proposed that the cyanopyrazole ring is more effective at interfering with helical structures than the cyanophenyl ring. Additionally, the core contact interaction may be initiated by Trp (W399) with cyanopyrazole ring. 

Although the scope of this work is limited to the conformational changes upon SARICA ligation within a single polypeptide, there is an intriguing connection between the previous experimental finding that SARICAs neutralize LLPS and the current finding of how SARICAs induce local conformational changes. To identify the mechanism of LLPS and study the LLPS driving forces (defined as the strong intermolecular interaction that is responsible for protein condensate formation and LLPS) for the intact AF1 domain, protein–protein interaction needs to be studied and parts of AF1 that form intermolecular interactions need to be identified. There are various studies on identifying the LLPS scaffold interactions and it is quite clear that not all LLPS use the same theme [66,67]. It is possible that nonspecific interaction leads to IDP condensation, but there is also possibility that the driving force is caused by specific protein–protein interaction of ordered structures, which is supported by studies in folding-upon-binding and amyloid formation [68,69,70,71]. 

Among the various hypotheses, one plausible scenario that is supported by our results on AR-AF1 region is that transient helical packing contributes to the LLPS driver in the form of a tertiary structure interaction. Further, we showed that SARICAs might disrupt helical structures, which could potentially interrupt the driver of the condensate. Future work on this topic could start with studying the LLPS driver of the AF1 domain directly, such as a study focusing on interactions between helical components and how ligands change the nature and/or the strength of the interaction.

## 4. Concluding Remarks

Small-molecule ligands can affect the collective behavior of IDP and, in the case of the AF1 region of the AR, the aggregate is neutralized when SARICA ligands are introduced. To understand the mechanism of ligand effects on IDR conformations, we constructed computer models of various versions of unmodified and ligand-attached peptide segments from the AF1 region. Through sampling of the conformations by molecular dynamics (MD) simulation and statistical analysis, we found that SARICAs that contain cyanopyrazole have a relatively strong interaction with the peptide segment 406C (especially with residue W399) and decrease the helical propensity of the peptide. On the other hand, ineffective LLPS neutralizers that contain a cyanophenyl ring have a weaker capability of destabilizing the helical structure. These structural and biophysical interaction differences can be a starting point for further inquiry towards understanding the mechanism of AR-AF1 condensation. It might provide insights on how neutralizers of other IDR aggregates of a similar nature should be designed.

## Figures and Tables

**Table 1:** The peptide systems constructed in this study. The short-hand notation indicates the system status as a combination of ligand identity, peptide identity (a 21-mer centered on a specific Cys residue) and starting conformations (E for extended and H for α-helical). Here, “4” is for “406”, and “3” for “327”, and “2” for “240”. The simulation length and solvent composition are also listed.

Notation	Center	S.C.	Ligand	Run Time	Ions	H_2_O#
XNN_4H	406	H	XNN	100 ns	1 Cl^−^	2701
XNN_4E	406	E	XNN	100 ns	1 Cl^−^	6898
NTD_4H	406	H	-	100 ns	1 Cl^−^	2731
NTD_4E	406	E	-	100 ns	1 Cl^−^	5570
XN0_4H	406	H	XN0	100 ns	1 Cl^−^	2687
XNB_4H	406	H	XNB	100 ns	1 Cl^−^	2886
XNN_3H	327	H	XNN	100 ns	1 Na^+^	2701
XNN_3E	327	E	XNN	50 ns	1 Na^+^	6880
NTD_3H	327	H	-	100 ns	1 Na^+^	2522
NTD_3E	327	E	-	100 ns	1 Na^+^	5460
XNN_2H	240	H	XNN	50 ns	-	2980
NTD_2H	240	H	-	100 ns	-	2774
NTD_2E	240	E	-	100 ns	-	3415
XEN_4H	406	H	XEN	50 ns	1 Cl^−^	2695

## Data Availability

Models and parameters of ligands used in this study and simulation trajectory data are available upon request.

## Supplementary Materials

The following supporting information can be downloaded at: https://www.mdpi.com/article/10.3390/biology12111442/s1, Figure S1: The full sequence of human androgen receptor; Figure S2: The PONDR score and an AlphaFold structure model; Figure S3: The SMILE codes of covalent binders used in this study; Figure S4: The dynamic evolution of helical formation percentage as a function of the simulation time.
