# Supplementary material for: Novel Covalent Modifier-Induced Local Conformational Changes within the Intrinsically Disordered Region of the Androgen Receptor
Source: Biology (Basel). 2023 Nov 17;12(11):1442. doi: 10.3390/biology12111442 (PMC10669190; doi:10.3390/biology12111442)
Supplement: Supplementary file 1 [file biology-12-01442-s001.zip › biology-2617224-supplementary.pdf]

**Supplementary Material for Novel Covalent Modifier Induced Local Conformational Changes within the Intrinsically Disordered Region of Androgen Receptor**

**Michael T. Harnish[1], Daniel Lopez[1], Corbin T. Morrison[1], Ramesh Narayanan[2], Elias J. Fernandez[1], and Tongye Shen[1]**

[1] Department of Biochemistry & Cellular and Molecular Biology, University of Tennessee, Knoxville, TN, 37996

[2] Department of Medicine, College of Medicine, University of Tennessee Health Science Center, Memphis, TN, 38103

MEVQLGLGRVYPRPPSKTYRGAFQNLFQSVREVIQNPGPRHPEAASAAPP
GASLLLLQQQQQQQQQQQQQQQQQQQQQQQETSPRQQQQQQGEDGSPQAH 100
RRGPTGYLVLDEEQQPSQPQSALECHPERGCVPEPGAAVAASKGLPQQLP
APPDEDDSAAPSTLSLLGPTFPGLSSCSADLKDILSEASTMQLLQQQQQE 200
AVSEGSSSGRAREASGAPTSSKDNYLGGT**STISDNAKELCKAVSVSMGLG**
VEALEHLSPGEQLRGDCMYAPLLGVPPAVRPTPCAPLAECKGSLLDDSAG 300
KSTEDTAEYSPFKGGY**TKGLEGESLGCSGSAAAGSSG**TLELPSTLSLYKS
GALDEAAAYQSRDYYNFPLALAGPPPPPPPPHPHARIKLENPLDY**GSAWA** 400
**AAAAQCRYGDLASLHG**AGAAGPGSGSPSAAASSSWHTLFTAEEGQLYGPC
GGGGGGGGGGGGGGGGGGGGGGGEAGAVAPYGYTRPPQGLAGQESDFTAP 500
DVWYPGGMVSRVPYPSPTCVKSEMGPWMDSYSGPYGDMRLETARDHVLPI
DYYFPPQKTCLICGDEASGCHYGALTCGSCKVFFKRAAEGKQKYLCASRN 600
DCTIDKFRRKNCPSCRLRKCYEAGMTLGARKLKKLGNLKLQEEGEASSTT
SPTEETTQKLTVSHIEGYECQPIFLNVLEAIEPGVVCAGHDNNQPDSFAA 700
LLSSLNELGERQLVHVVKWAKALPGFRNLHVDDQMAVIQYSWMGLMVFAM
GWRSFTNVNSRMLYFAPDLVFNEYRMHKSRMYSQCVRMRHLSQEFGWLQI 800
TPQEFLCMKALLLFSIIPVDGLKNQKFFDELRMNYIKELDRIIACKRKNP
TSCSRRFYQLTKLLDSVQPIARELHQFTFDLLIKSHMVSVDFPEMMAEII 900
SVQVPKILSGKVKPIYFHTQ

Color Labels: NTD-I; NTD-II; DBD; Linker; LBD; CTD; AF1 (141-487);

Uniprot ID P10275 Human androgen aeceptor (920 a.a.)

**Figure S1**: The full sequence of human androgen receptor. The underlined region is largely disordered AF1. Three 21-residue peptide segments examined in this work are shown in a bold font. Each centered on a CYS (240, 327, and 406) that may be modified by a covalent binder in this study.

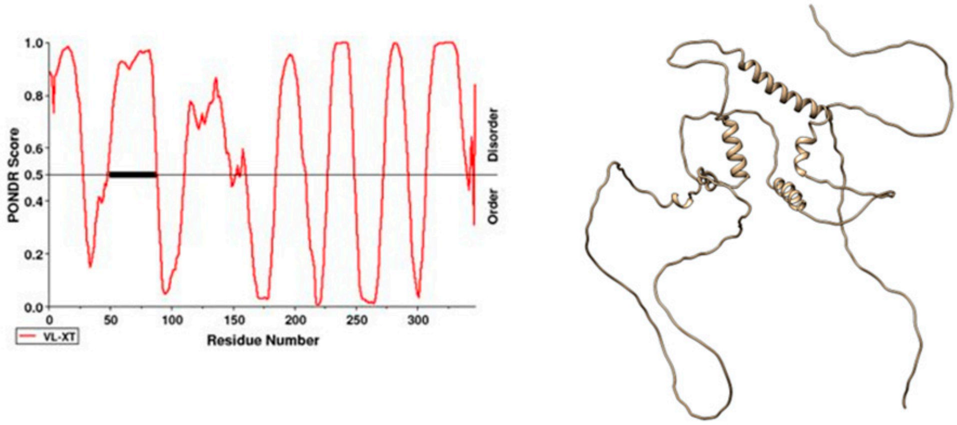


**Figure S2**: The PONDR score and an AlphaFold structure model. Left: The PONDR score of the largely disordered AF1 plotted using internal index (+141 for converting to standard hAR residue index). Right: The AlphaFold2 present this AF1 as several transient helices loosely interacting with each other.

**XNN:**

COC(=O)\C(CO[C@@](C)(C[N]1C=C(C=N1)C#N)C(=O)
NC2=CC(=C(N=C2)C#N)C(F)(F)F)=C\SC[C@H](N)C(O)=O

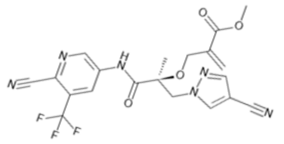

**XNB:**

COC(=O)C(COC(C)CC1=CC=C(C=C1)C#N)C(=O)
NC2=CC(=C(N=C2)C#N)C(F)(F)F)CSC[C@H](N)C(O)=O

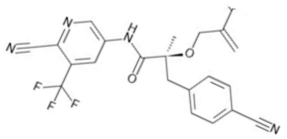

**XN0:**

N[C@@H](CSCC(C[N]1C=C(C=N1)C#N)C(=O)
NC2=CC(=C(N=C2)C#N)C(F)(F)F)C(O)=O

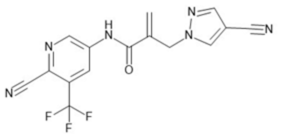

**XEN:**

N[C@@H](CSC[C@H](COC1=CC=C(C=C1)C#N)C(=O)
OC2=CC=C(C=C2)C#N)C(O)=O

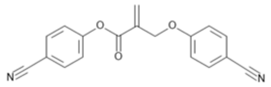

**Figure S3**: The SMILE codes of covalent binders used in this study: XNN, XNB, XN0, and XEN.

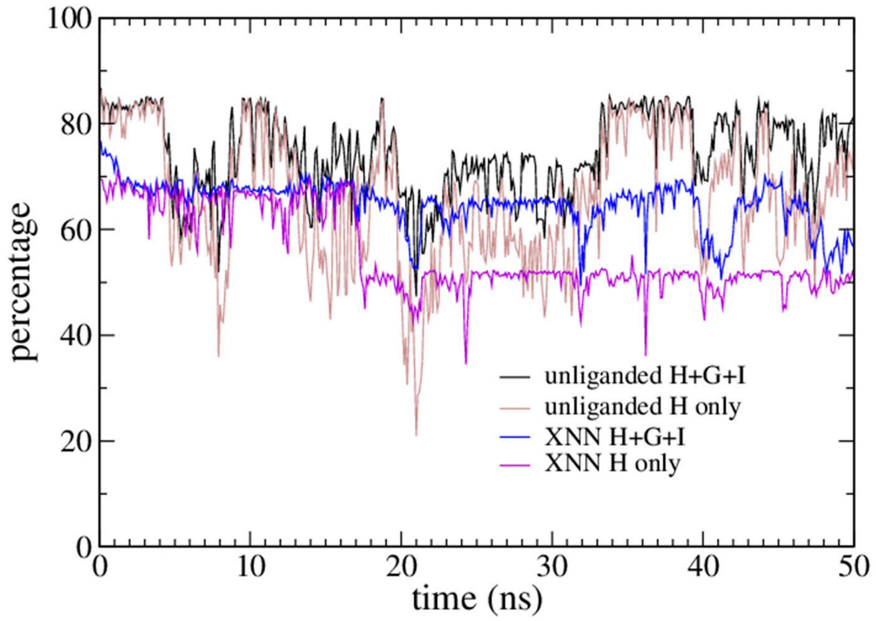


**Figure S4**: The dynamic evolution of helical formation percentage as a function of the simulation time. For H only, only α-helix is assigned a value of 1 and all other states defined in DSSP are considered 0. For H+G+I, all three helical states (α-, $3_{10}$- and π-helical) are assigned a value of 1 and all of the remaining states (E, B, S, T, and C) are 0. The average does not include two terminal capping residues.
